# Genome-wide analysis of the metallothionein gene family in cassava reveals its role in response to physiological stress through the regulation of reactive oxygen species

**DOI:** 10.1186/s12870-023-04174-2

**Published:** 2023-04-28

**Authors:** Yanyan Ma, Maofu Xue, Xiaofei Zhang, Songbi Chen

**Affiliations:** 1 School of Life Sciences, Hainan University, Haikou, 570228 China; 2 Tropical Crops Genetic Resources Institute, Chinese Academy of Tropical Agricultural Sciences/Key Laboratory of Ministry of Agriculture for Germplasm Resources Conservation and Utilization of Cassava, Haikou, 571101 China; 3 Alliance of Bioversity International and CIAT, Cali, 763537 Colombia

**Keywords:** Cassava, *MT* genes, Gene expression, Abiotic stress, ROS

## Abstract

**Background:**

Cassava (*Manihot esculenta* Crantz) is widely planted in tropical and several subtropical regions in which drought, high temperatures, and other abiotic stresses occur. Metallothionein (MT) is a group of conjugated proteins with small molecular weight and rich in cysteine. These proteins play a substantial role in response to physiological stress through the regulation of reactive oxygen species (ROS). However, the biological functions of *MT* genes in cassava are unknown.

**Results:**

A total of 10 *MeMT* genes were identified in the cassava genome. The *MeMTs* were divided into 3 groups (Types 2–4) based on the contents and distribution of Cys residues. The *MeMTs* exhibited tissue-specific expression and located on 7 chromosomes. The *MeMT* promoters contain some hormones regulatory and stresses responsiveness elements. *MeMTs* were upregulated under hydrogen peroxide (H_2_O_2_) treatment and in respond to post-harvest physiological deterioration (PPD). The results were consistent with defense-responsive cis-acting elements in the *MeMT* promoters. Further, four of *MeMTs* were selected and silenced by using the virus-induced gene silencing (VIGS) method to evaluate their functional characterization. The results of gene-silenced cassava suggest that *MeMTs* are involved in oxidative stress resistance, as ROS scavengers.

**Conclusion:**

We identified the 10 *MeMT* genes, and explore their evolutionary relationship, conserved motif, and tissue-specific expression. The expression profiles of *MeMT*s under three kinds of abiotic stresses (wounding, low-temperature, and H_2_O_2_) and during PPD were analyzed. The tissue-specific expression and the response to abiotic stresses revealed the role of *MT* in plant growth and development. Furthermore, silenced expression of *MeMTs* in cassava leaves decreased its tolerance to ROS, consistent with its predicted role as ROS scavengers. In summary, our results suggest an important role of *MeMTs* in response to physiological stress as well as species adaptation via the regulation of ROS homeostasis.

**Supplementary Information:**

The online version contains supplementary material available at 10.1186/s12870-023-04174-2.

## Background

Metallothionein (MT) is a group of conjugated proteins with small molecular weight and rich in cysteine. These proteins play a substantial role in response to physiological stress through the regulation of ROS [1]. Since the discovery of the first plant MT protein (*EcMT)* in wheat in 1987 [2], more MT genomic sequences from various species have been identified [3–7]. *Arabidopsis thaliana* and *Oryza sativa* have 7 and 14 *MT* genes respectively. *AtMT2a* has been demonstrated to influence ROS balance under oxidative stress induced by low temperature [8]. Moreover, an in vitro experiment revealed that recombinant OsMT2b protein could scavenge superoxide and hydroxyl radicals [9]. Previous studies showed that plant *MT* played a crucial role in the detoxification of some heavy metals, for example, *OsMT1b* and *OsMT2*c had an impact on metal detoxification, in which they enhanced the ability to detoxify Cr in rice [10].

Cassava is the sixth largest staple food crop grown in tropical and subtropical regions, feeding about 1 billion people globally [11]. However, cassava growth and development experience various biotic and abiotic stresses, especially wounding and low-temperature injury, which not only affects the growth of cassava, but also reduces its quality and yield [12–14]. Studies have shown that the accumulation and rapid burst of ROS in cassava storage roots are the main causes of PPD [15].

In this study, 10 *MeMTs* were identified in the cassava genome. A phylogenetic tree was constructed to show their evolutionary relationship. The *MeMTs* had different expression patterns under various abiotic stresses. Finally, *MeMTs* were silenced by VIGS to evaluate their roles in conferring tolerance against ROS [16]. Our results will not only be helpful to understand the molecular mechanism in cassava in response to stresses, but also provide a clue to improve cassava varieties.

## Results

### Identification and Cys residues analysis of ***MeMTs***

**Fig. 1:**
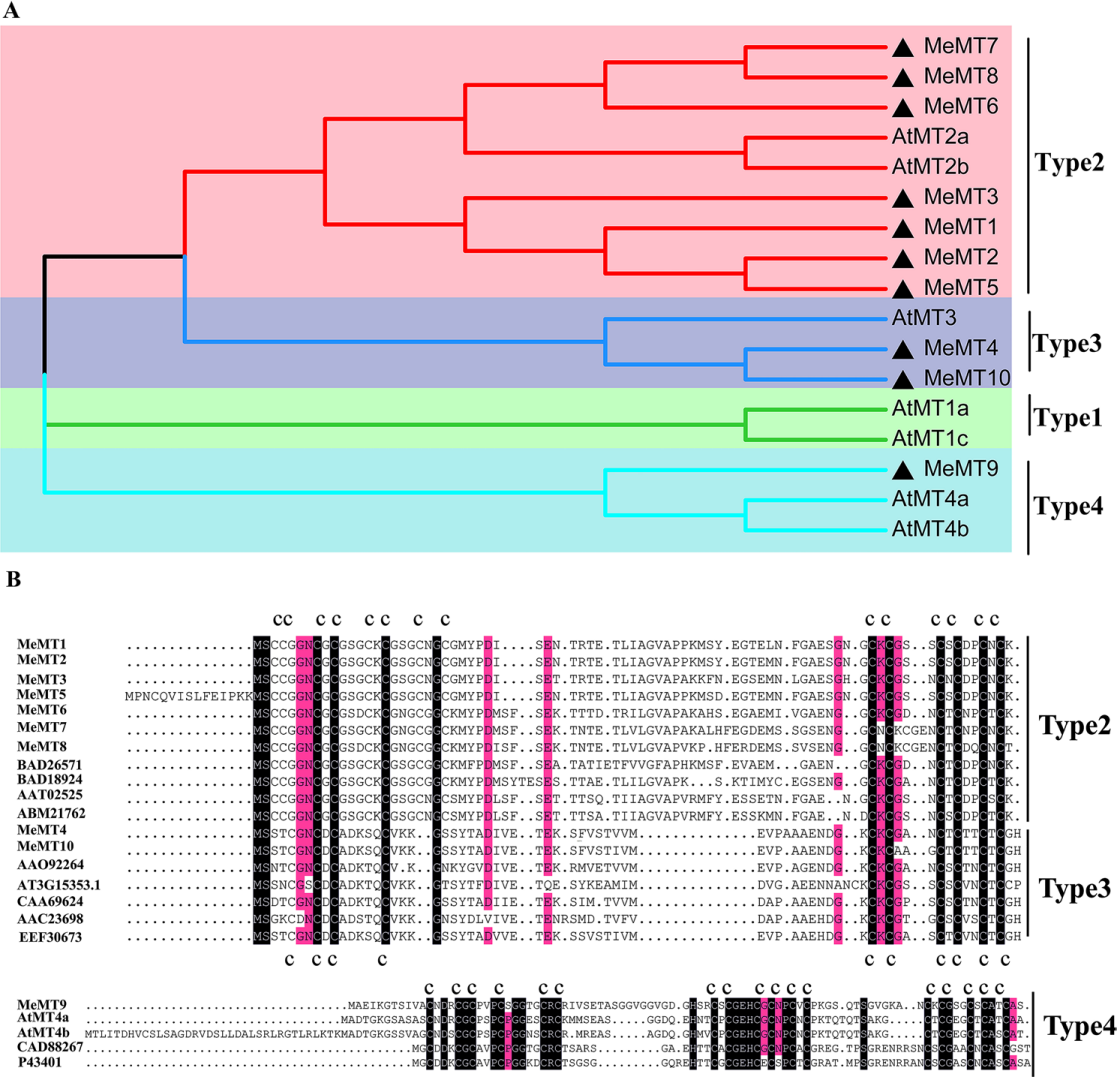
Phylogenetic tree and multiple sequence alignment of cassava MTs. **(A)** Phylogenetic tree consisting of cassava and *Arabidopsis* MT proteins. The MeMTs are indicated by black triangles. Different color regions represent different types. **(B)** Comparison of the deduced aa of MeMTs with their homologs from other plant species. The letter C denotes Cys residues

The 10 *MeMT* genes from *MeMT1* to *MeMT10* were identified (Table 1, Additional file 1: Table S1, Table S2). The open reading frames (ORFs) of the 10 *MeMT*s ranged from 201 to 303 bp in length, encoding proteins comprising 66 amino acids (aa) to 93 aa. The MeMT10 protein had the lowest molecular weight (MW) at 6.8 kDa, and the highest MW was recorded for MeMT2—10.335 kDa. The contents of Cys residues for the 10 *MeMTs* ranged from 14.9 to 18.7%. MeMT4 and MeMT10 have 10 Cys residues, with 4 in the N-terminus and 6 in the C-terminus, characteristic of a typical Type 3 plant MT proteins. MeMT9 has 17 Cys residues, with 6 in the N-terminus, 5 in the C-terminus, 6 in the middle of both the N-terminus and the C-terminus, which is characteristic of a typical Type 4 plant MT proteins. The other 7 MeMTs preserve the characteristics of Type 2 plant proteins. They contain 14 Cys residues, which are split into two Cys-rich domains by a Cys-free spacer of around 40 amino acids, with 8 in the N- and 6 in the C-terminus. (Fig. 1B). The 10 *MeMTs* are grouped into Type 2, Type 3 *(MeMT4, MeMT10*) and Type 4 (*MeMT9*) according to the sequences of their *Arabidopsis* homologs (Fig. 1A).

**Table 1:** Characteristics of *MeMT* genes

Gene name	Gene ID	ORF (bp)	ORF (aa)	MW^a^ (kDa)	pI	Cys number	Cys content (%)	Spacer^d^ (aa)
MeMT1	MANES_01G174200	237	78	7.8	4.77	8 + 6^b^	18.0	40
MeMT2	MANES_01G174400	303	100	10.0	5.22	8 + 6	14.0	40
MeMT3	MANES_02G133200	237	78	7.6	5.63	8 + 6	18.0	40
MeMT4	MANES_03G110500	204	67	6.9	5.08	4 + 6	15.0	35
MeMT5	MANES_07G091300	282	93	9.5	4.89	8 + 6	15.0	40
MeMT6	MANES_08G079000	240	79	7.9	5.63	8 + 6	17.7	41
MeMT7	MANES_08G079100	249	82	8.3	5.16	8 + 6	17.0	42
MeMT8	MANES_08G079200	246	81	8.4	4.68	8 + 6	17.2	41
MeMT9	MANES_11G110300	276	91	8.7	8.13	6 + 6 + 5^c^	18.6	20/13
MeMT10	MANES_15G086700	201	66	6.8	5.08	4 + 6	15.0	34

^a^MW Molecular weight; ^b^ (8 + 6) the Cys number with 8 in the N- and 6 in the C-terminus; ^c^ (6 + 6 + 5) the Cys number with 6 in the N-terminus, 5 in the C-terminus, 6 in the middle of both the N-terminus and the C-terminus; ^d^Spacer the number of aa between Cys cluster

### Phylogenetic analysis of the ***MeMTs***

**Fig. 2:**
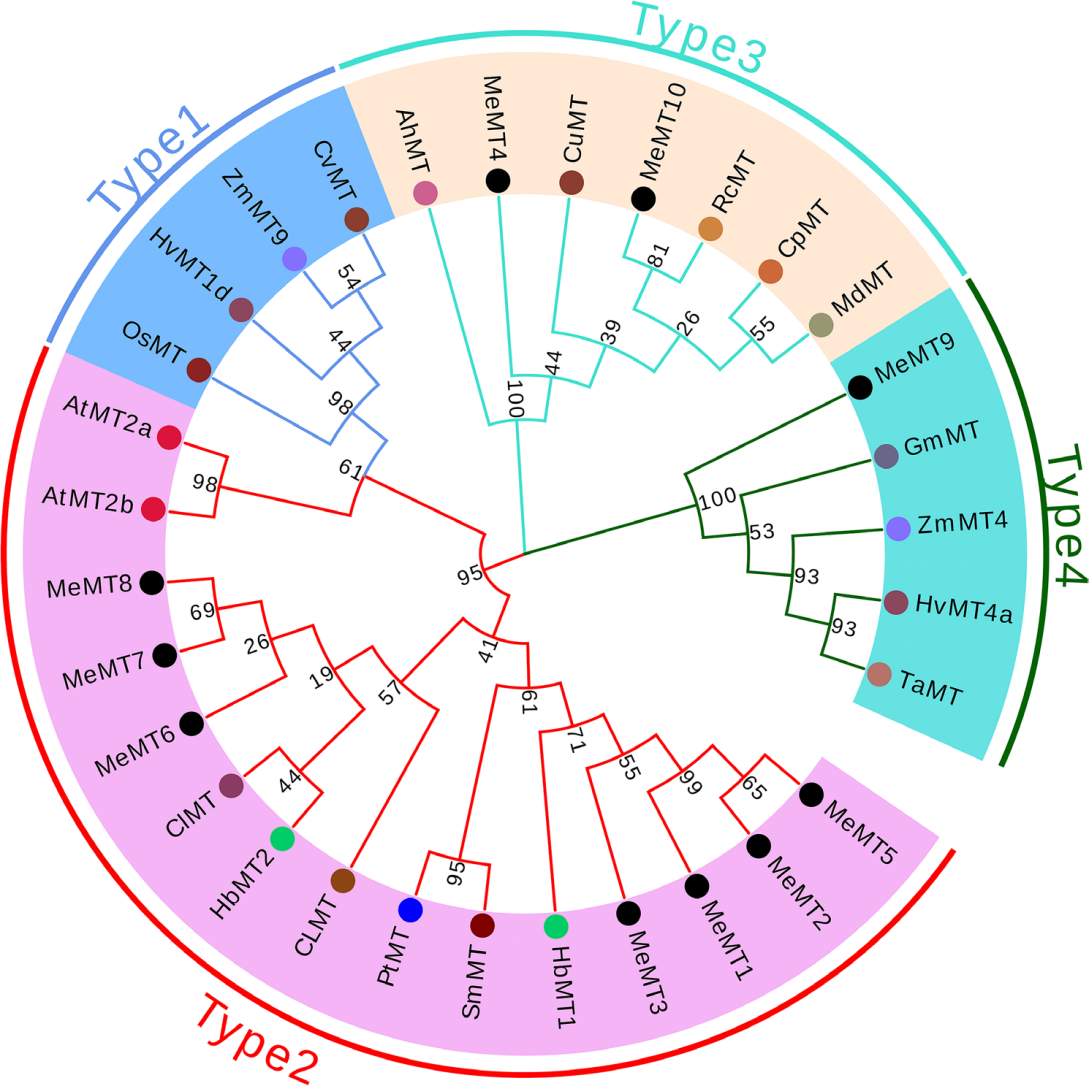
Phylogenetic analysis of 31 *MT* gene-encoding proteins. The blank circles represented *MeMT* genes. The different color circles represent different species, respectively.

To learn more about the functions and evolutionary history of the *MeMTs*, a total of 31 *MT* genes (Additional file 1: Table S3) from *Glycine max*, *Malus domestica*, *Arachis hypogaea*, *Populus trichocarpa* × *Populus deltoides*, *Oryza sativa*, *Salix matsudana*, *Hevea brasiliensis*, *Arabidopsis*, *Codonopsis lanceolata*, *Citrullus lanatus*, *Chloris virgata*, *Triticum aestivum*, *Zea mays*, *Carica papaya, Hordeum vulgare*, and *Ricinus communis* were used to construct a neighbor-joining (NJ) phylogenetic tree (Fig. 2). The results revealed that the 31 *MT* genes may be classified into four subgroups, which were named Types 1–4, as shown in Fig. 2. The *MeMT* family genes are present in the Type 2–4 subgroup and are not present in the Type 1 subgroup, indicating that there were no genes whose structure and function were similar to those of *OsMT* in cassava.

**Fig. 3:**
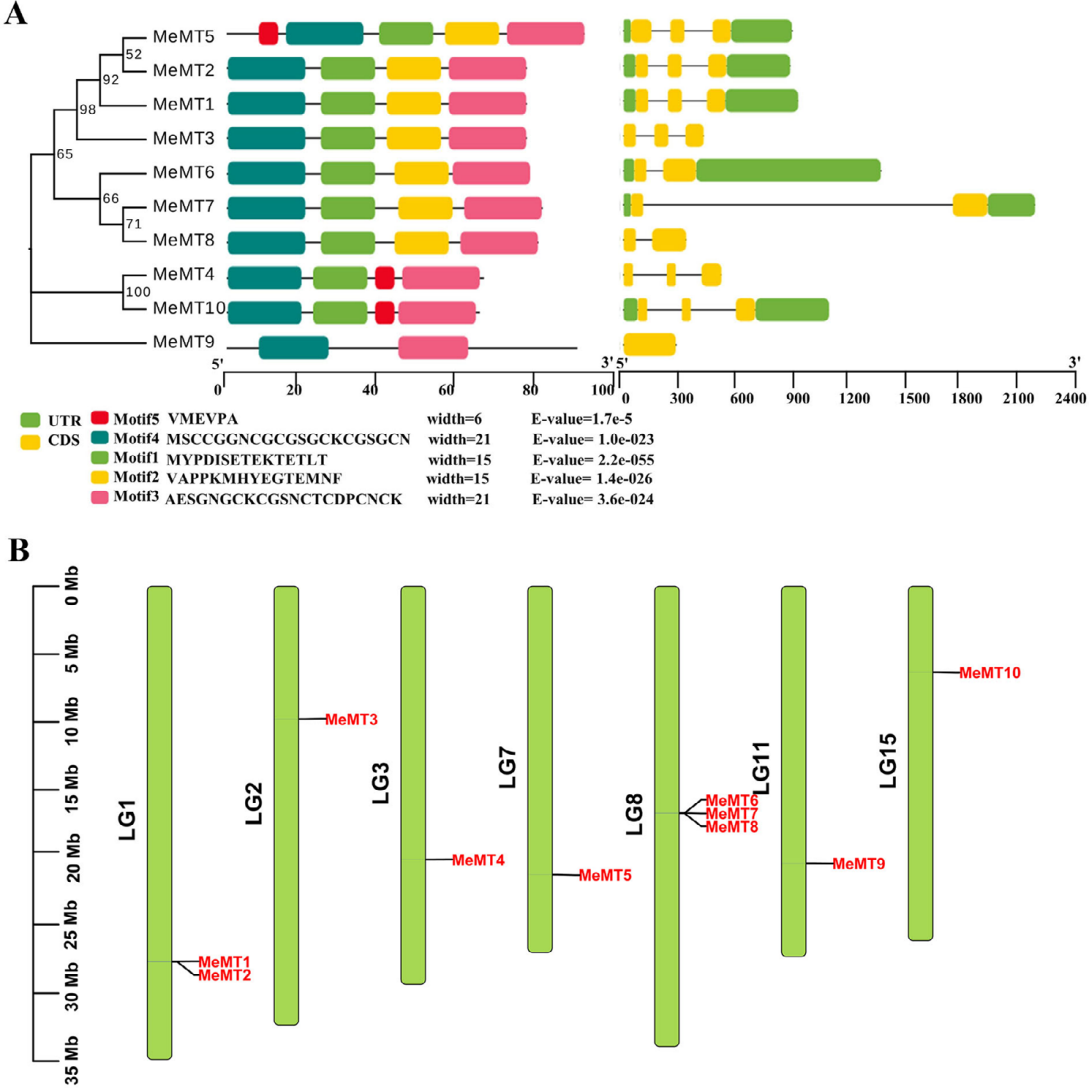
Gene structure, conserved protein motifs and chromosomal distribution of *MeMTs*. **(A)** The phylogenetic tree comprising 10 *MeMT* genes was generated via the MEGA 7.0 program, which included the exon‒intron structures of the 10 *MeMT* genes. Five patterns of conserved protein motifs were depicted in different colored boxes. **(B)** Chromosome distribution of *MeMT* genes. 10 *MeMT* genes were mapped onto the 7 Chromosomes of cassava

The Type 1 and Type 2 subfamily members were closely related to each other, which suggesting that the members in Type 2 subfamily evolved from the members in Type 1 subfamily [17]. The *MeMT* genes were clustered together in the phylogenetic tree as shown in Fig. 2. For example, *MeMT1* and *MeMT2* clustered together onto chromosome LG_01 (Fig. 3B), and they also clustered together into the Type 2 group in the phylogenetic tree. These phenomena indicated that *MeMT* genes may have originated from gene duplication.

### Gene structure, conserved protein motifs and chromosomal distribution of ***MeMT*** genes

The gene structure of the *MeMT*s was investigated to have a better understanding of the evolution of the *MeMT* family in cassava. The *MeMTs* possessed relatively simple gene structures. Among the *MeMT* genes, the Type 3 subfamily members had two introns, and the Type 4 subfamily members had no intron (Fig. 3A). The gene structures of type 2 were slightly diverse, in which *MeMT6, MeMT7* and *MeMT8* had one intron and other *MeMTs* have two introns.

To gain insight into the functional regions of MeMT proteins, we revealed the conserved motifs among the 10 MeMT proteins, and identified 5 conserved motifs (Fig. 3A). Members of the same group shared patterns, implying that these proteins perform similar functions. The most conserved motifs 3 and 4 were exhibited in all MeMT proteins. Motifs 4, 1, 5 and 3 were presented in the Type 3 group members. The members of Type 2 group had the greatest number of motifs. Type 4 members exclusively contained the highly conserved motifs 3 and 4 (Fig. 3A). Especially, the motif 5 were unique to Type 2/3. The motifs exist in certain groups, which may be related to specific biological functions.

To clearly understand the chromosome distribution of the *MeMT* gene family members, we constructed a chromosome distribution map of 10 cassava *MT* genes (Fig. 3B). We found that 10 *MeMTs* were randomly distributed on 7 chromosomes (Chr 2, Chr 3, Chr 7, Chr 11, Chr 15). Most *MeMT* genes are located on an independent chromosome except in the case of Chr 1, which contains 2 *MeMT* genes (*MeMT1* and *MeMT2*). Chr 8 has the most genes—a total of three (*MeMT6, MeMT7* and *MeMT8*). Notably, three of them (*MeMT1*, *MeMT2* and *MeMT3*) were distributed in the reverse direction, while the other 7 members were distributed in a forward direction.

### Duplications and synteny analysis of the ***MeMT*** genes

**Fig. 4:**
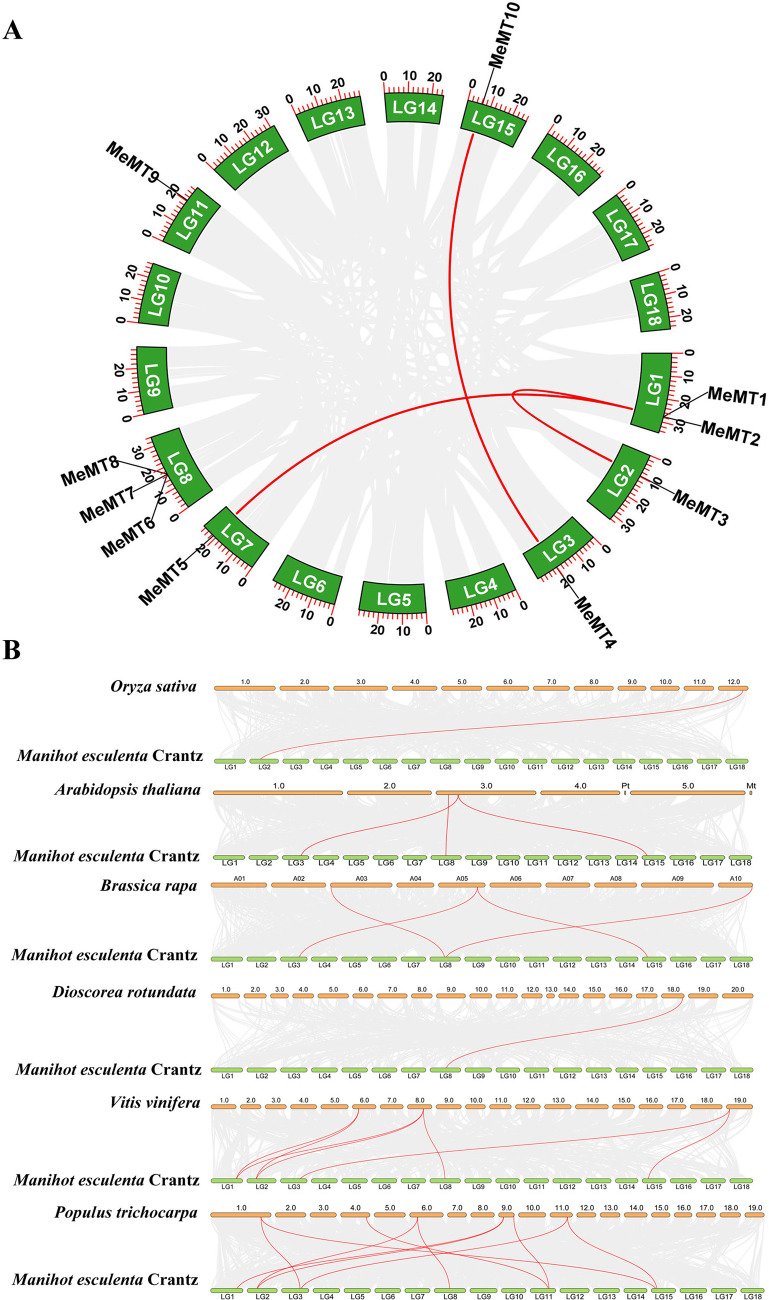
Duplications and synteny analysis of *MeMT* genes. **(A)** Duplication analysis of *MTs* in cassava. Duplicate blocks are shown by gray lines, and duplicate *MeMT* gene pairs are shown by red lines. **(B)** syntenic maps between cassava and other species. The syntenic *MT* gene pairs were represented with the red lines

To reveal the expansion process of the *MeMTs* members, we conducted an intragenomic synteny analysis among the genes. Segmental and tandem duplications are considered to be the main reasons leading to gene family expansion in plants [18]. As shown in Fig. 4A, some genes (*MeMT1/MeMT2, MeMT7/MeMT6*, *MeMT7/MeMT8*) were adjacent to other and located sequentially in tandem on chromosomes 1 and 8, suggesting that these genes might have expanded via tandem duplication. In addition to the tandem duplication, 6 *MeMT* genes (*MeMT1/MeMT3, MeMT4/MeMT10 and MeMT1/MeMT5)* were located in segmental duplication blocks (Fig. 4A). Based on the nonsynonymous (Ka) and synonymous (Ks) of each duplicated *MeMT* gene pair, the Ka/Ks value of each gene pair was also calculated (Additional file 1: Table S4). *MeMT1/MeMT3* and *MeMT4/MeMT10* had Ka/Ks values that were < 1; specifically, the values were 0.1460 and 0.0916, respectively. However, the Ka/Ks of *MeMT1/MeMT5* was NaN. These results suggested that those *MeMTs* were possibly subjected to negative selection.

To further explore the syntenic relationships of the *MeMT* family members among other plants species, syntenic maps were constructed to explore homology (Fig. 4B), which included four dicots (*Populus trichocarpa*, *Brassica rapa*, *Vitis vinifera* and *Arabidopsis thaliana*) and two monocots (*Oryza sativa* and *Dioscorea rotundata*). 10 *MeMT* genes showed a syntenic relationship with their homologs in *(A) thaliana* (2), *O. sativa* (1), *P. trichocarpa* (6), *(B) rapa* (3), *D. rotundata* (1) and *V. vinifera* (3) (Additional file 1: Table S5). The most *MeMT* homologs were presented in *P. trichocarpa*, while the monocots rice (*O. sativa*) and yam (*D. rotundata*) exhibited the fewest homologs. Taken together, the results may be explained by the closer phylogenetic relationships among the dicots relative to the monocots. In addition, many *MeMT* genes were identified as putative orthologs of a single *AtMT* gene, suggesting that the expansion of *MeMT* genes may have occurred after that in *A. thaliana* during evolution. For example, both *MeMT4* and *MeMT10* are orthologs of *AtMT3* gene. Some continuous collinear gene pairs were found in cassava and in other species, such as *MeMT4/AtMt3* and *MeMT4/BraMt5*, which suggested that these genes may have been involved in the evolution of the *MeMT* gene family.

### ***cis***-elements analysis and expression patterns of ***MeMT*** genes in different tissues

**Fig. 5:**
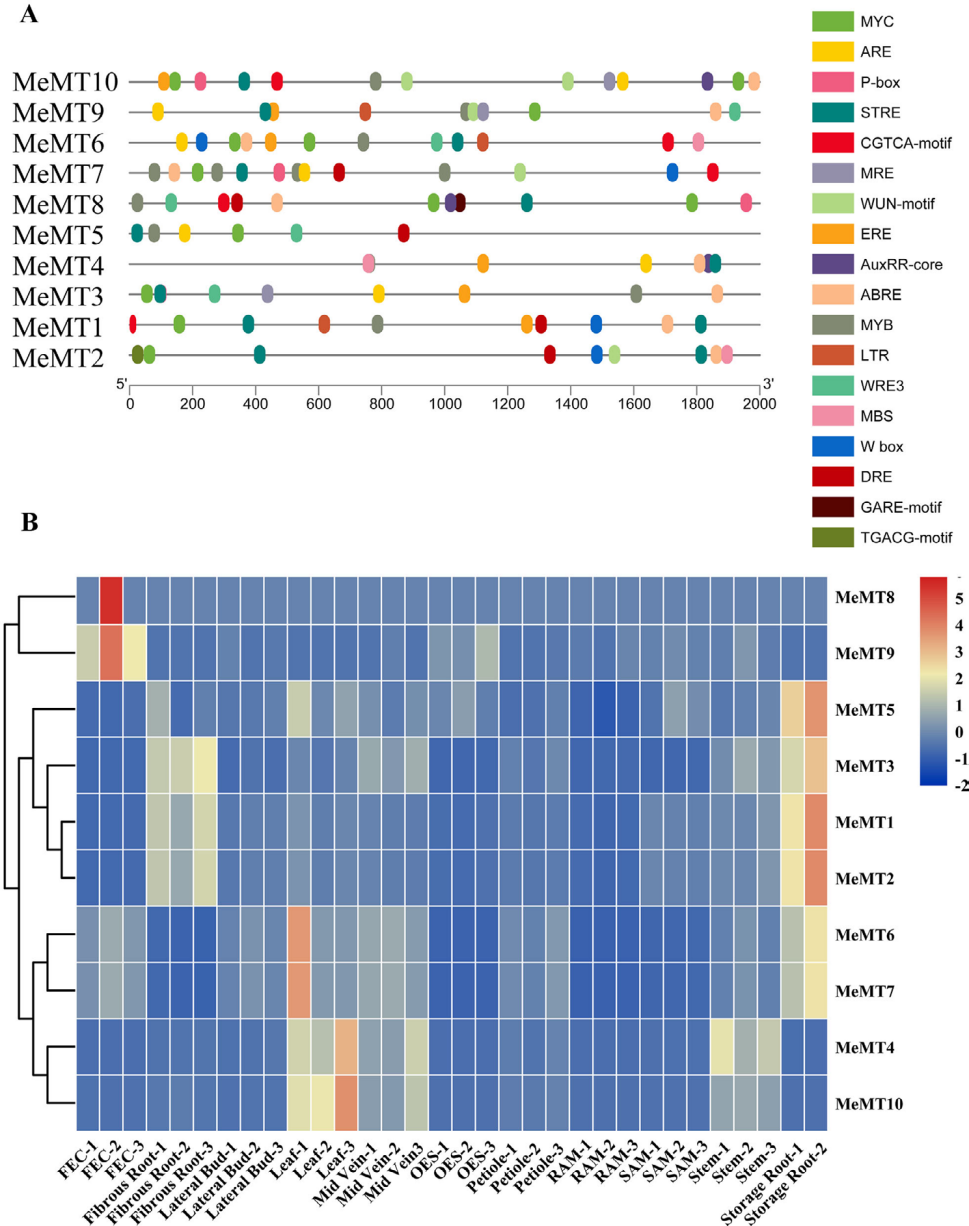
Regulatory *cis*-acting elements analysis and expression patterns of *MeMT* genes in different tissues. **(A)** Predicted *cis*-acting elements in *MeMT* promoters. Different *cis*-acting elements are depicted by different colors boxes. **(B)** Expression patterns of cassava *MeMT* genes in various tissues. The transcript levels are depicted by different colors on the scale. The blue and red colors represented low and high expression levels, respectively

To explore the potential *cis*-elements involved in biotic and abiotic stresses, the 2 kb upstream sequences of *MeMTs* were programmed in PlantCARE. All of the *cis*-elements may be relevant to hormones regulation and stress response (Fig. 5A, Additional file 1: Table S6). The ABREs and TGACG motif-containing element were correlated with ABA response and MeJA response, which widely spread in *MeMT* genes. Most of the stress responsive elements were correlated with drought response, low-temperature responsiveness and wound response. LTR elements (low-temperature responsiveness) were found in *MeMT9*, *MeMT6* and *MeMT1*, and WRE3 and WUN motifs (wounds) were found in all the *MeMT* genes except *MeMT1*. It is inferred that under different growth statuses and environmental conditions, *MeMT* genes could function independently or synergistically to ensure plant normal growth and development.

To explore the expression patterns of the *MeMT* gene family members in different tissues, we used transcriptome profiling data obtained via RNA sequencing (RNA-seq) to analyze *MeMT* genes expression in different cassava tissues (leaf, midrib, petiole, stem, lateral bud, stem apical meristem (SAM), storage root, fibrous root, root apical meristem (RAM), organized embryogenic structures and friable embryogenic callus (FEC) tissues) (Additional file 1: Table S7). The results showed that all *MeMTs* were expressed in at least one tissue (Fig. 5B). *MeMT9* and *MeMT8* exhibited relatively high expression in FEC tissue; *MeMT4* and *MeMT10* were highly expressed in the stems, leaves and midribs; and the others exhibited relatively high expression in the roots (fibrous roots and storage roots).

### Expression patterns of ***MeMT*** genes in response to different abiotic stresses

**Fig. 6:**
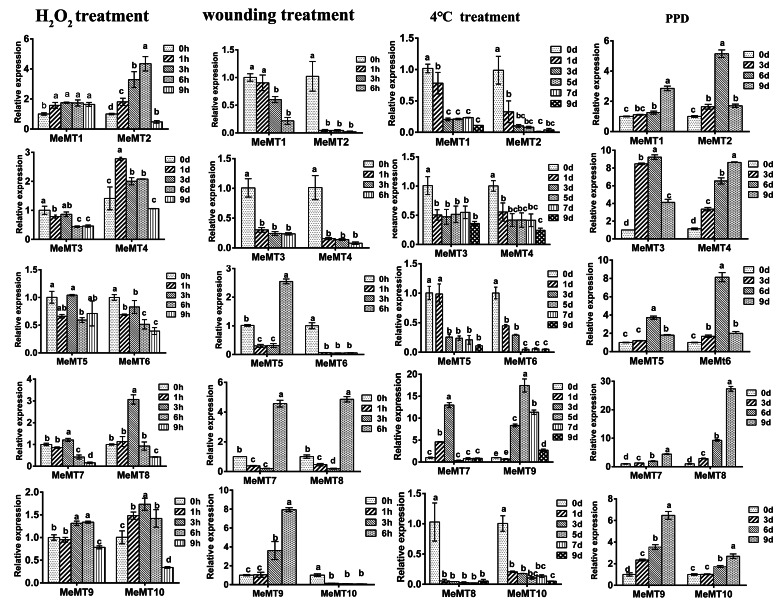
Expression profiles of *MeMT* genes in leaves of plants under abiotic stress and PPD.

To measure the transcript levels of *MeMT* genes in cassava in response to different abiotic stresses (wounding, low-temperature, and H_2_O_2_) and PPD, 10 *MeMT* genes of different types were subjected to qRT‒PCR (Fig. 6).

Under low-temperature treatment (4 ℃), the expression of *MeMT9* and *MeMT7* sharply rose and peaked at 5 d and 3 d, respectively, after which it decreased significantly, whereas the other eight *MeMT* genes showed down regulated expression at all the treatment timepoints. In particular, *MeMT8* expression was significantly induced at 1 d but did not display obvious trends for 3 ~ 9d. *MeMT5*, *MeMT7*, *MeMT8*, and *MeMT9* all showed considerable elevation under the wounding treatment at 6 h, but the expression of the other genes decreased at all treatment time points. The expression levels of *MeMT5, MeMT8, MeMT9*, and *MeMT7* were more than twofold higher at 6 h after wounding, indicating their possible function in wound-related signaling. Under H_2_O_2_ treatment, *MeMT2* and *MeMT4* showed significant upregulation at 6 and 1 h after treatment, respectively. The other 8 genes were upregulated at 3 h. *MeMT5* was strongly repressed at all the treatment times. During PPD in cassava, the transcript levels of 4 *MeMTs* (*MeMT6*, *MeMT5*, *MeMT2*, *MeMT3*) increased during early PPD progression but decreased during later PPD progression. Moreover, only the transcript levels of *MeMT3* significantly increased at the early stage (3 d). The expression of the other six *MeMTs* was upregulated at different stages and peaked at the last stage (9 d).

Together, these results indicated that most of the *MeMTs* could be significantly upregulated in response to H_2_O_2_ treatment and PPD, but that their expression was only slightly affected under low-temperature and wounding treatment, suggesting that *MeMTs* may participate in multiple signal transduction pathways in cassava.

### ***MeMTs*** regulate oxidative stress resistance in cassava

**Fig. 7:**
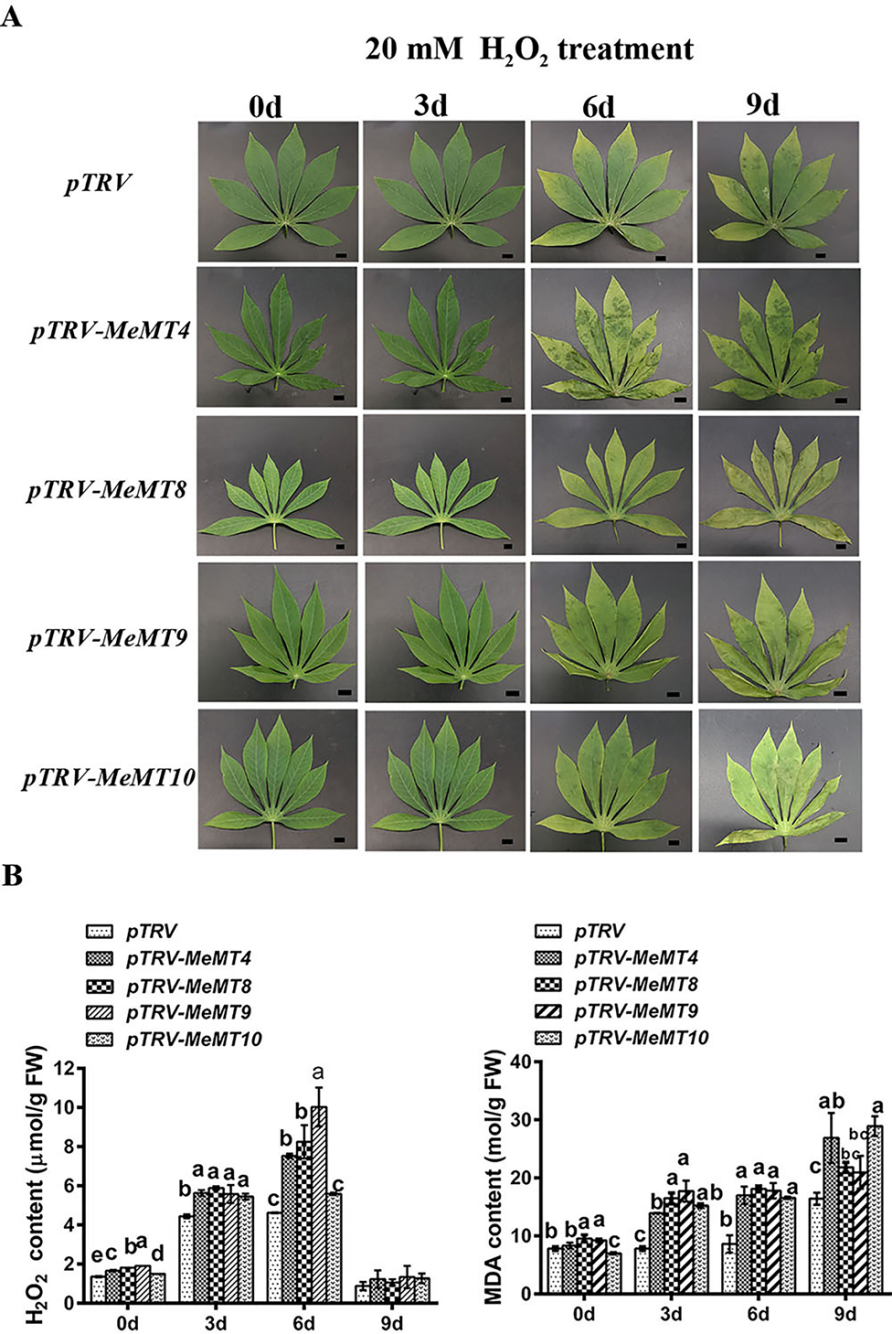
*MeMTs* regulate oxidative stress resistance in cassava. **(A)** The phenotype of *MeMT*-silenced cassava leaves under H_2_O_2_ treatment. Scale bar: 1 cm. (**B)** Quantification of the H_2_O_2_ content and MDA content in *MeMT*-silenced cassava leaves under H_2_O_2_ treatment.

Considering the significant upregulated expression in response to H_2_O_2_ treatment, *MeMTs* might pay a role in the oxidative stress response. To further elucidate the oxidative stress response, 4 *MeMTs* (*MeMT4, MeMT8, MeMT9* and *MeMT10*) were silenced by VIGS, which was subsequently verified by qRT‒PCR. As shown in Fig. 7, compared with the control cassava leaves, the *MeMT*-silenced cassava leaves were more sensitive to 20 mM H_2_O_2_ treatment. After H_2_O_2_ treatment for 3 h, compared with the control cassava, the silenced cassava presented higher ROS levels (H_2_O_2_ content) and severe cell damage, as reflected by the malondialdehyde (MDA) content. Overall, the *MeMT*-silenced cassava plants produced more H_2_O_2_ and had a lower ROS scavenging ability than did the control cassava plants. These results confirm that *MeMTs* are involved in the regulation of oxidative stress resistance in cassava.

**Fig. 8:**
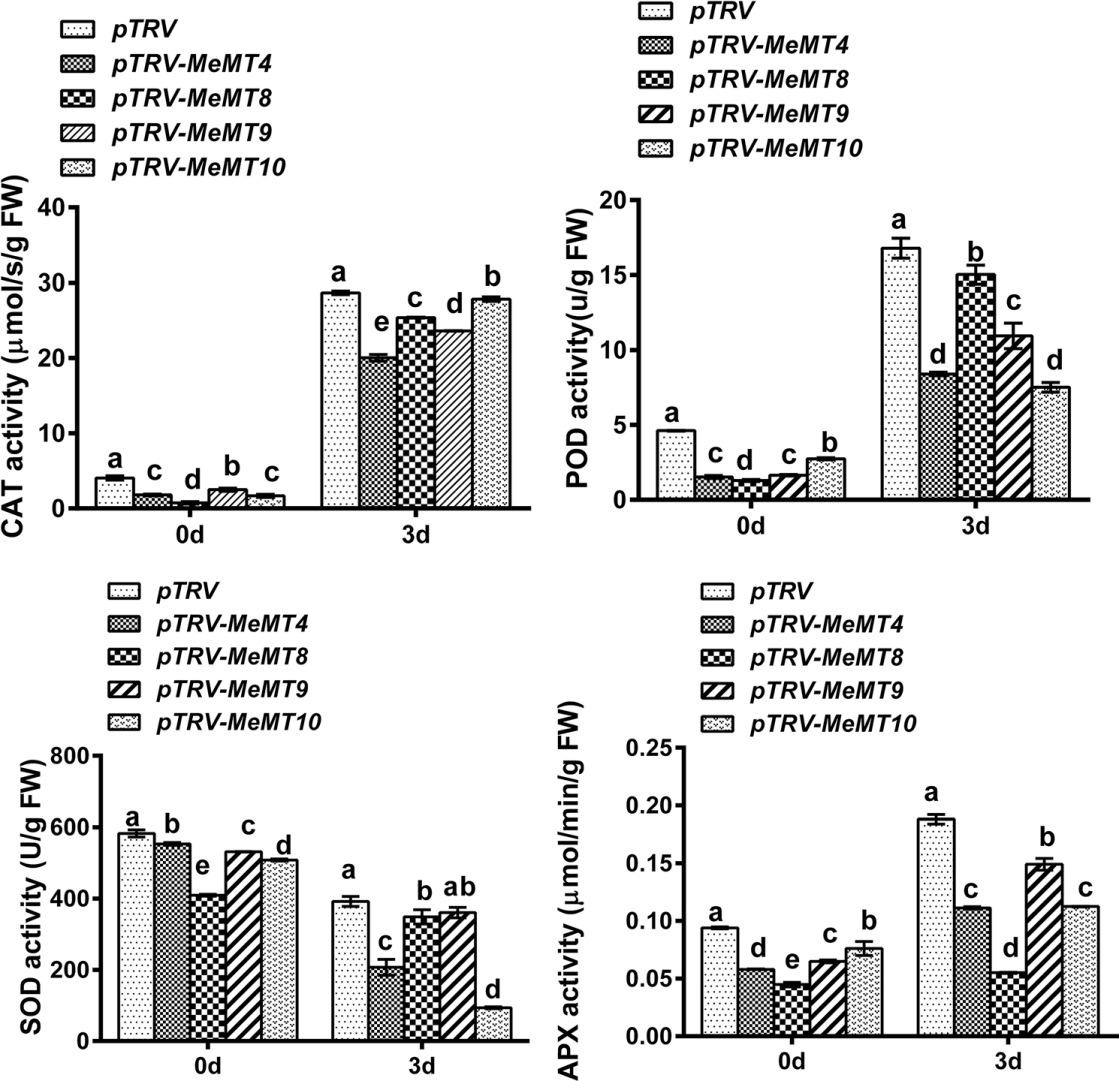
The enzymatic activities (CAT, POD, SOD, APX) of *MeMT*-silenced cassava leaves under H_2_O_2_ treatment

To explore how *MeMTs* affect ROS accumulation and the corresponding oxidative stress resistance in cassava, we analyzed the activities of four major antioxidant enzymes—catalase (CAT), peroxidase (POD), superoxide dismutase (SOD), and ascorbate peroxidase (APX)—in *MeMT*-silenced cassava leaves (Fig. 8). After H_2_O_2_ treatment, the CAT activity, POD activity, SOD activity and APX activity in four transgenic lines decreased compared with those in the control cassava plants. And the CAT activity, POD activity and APX activity were higher than that in the controls (0 d), which consistent with the changes of H_2_O_2_ concentration. However, after H_2_O_2_ treatment, the SOD activity was lower than that in the controls (0 d). These results seem to indicate that a high SOD activity needed to quench the increased of H_2_O_2_ content. This suggests that SOD would constitute the first line of defense against ROS and might be utilized as a signal molecule to get leaves ready for H_2_O_2_ before it arrives.

## Discussion

### Number and roles of ***MT*** genes

Previous studies have revealed 7, 11 and 10 *MT* genes in *Arabidopsis thaliana*, *Oryza sativa*, and *Helianthus annuus*, respectively[19–21]. The number of *MTs* in a given plant species is independent of its genome size, regardless of whether it is a dicot or monocot [1]. Based on the distribution of Cys residues in the N- and C-terminal regions of their encoded proteins, plant *MTs* are classified into four types: types 1, 2, 3 and 4 [22, 23]. The *MT* family in *Arabidopsis* consists of four types of *MTs* [24, 25]. Interestingly, the 10 *MeMTs* are only grouped into three types (type 2, type 3 and type 4) in according with their *Arabidopsis* homologs (Fig. 1A). One question about *MTs* is whether all four types are capable of functioning as metal chelators or ROS scavengers. Research has shown that, in *Arabidopsis*, all four types of *MTs* are capable of functioning as metal chelators, and *MT-1a, MT-2a, MT-2b*, and *MT-3* likely function as copper-binding *MTs*, whereas *MT-4a* and *− 4b* are more likely to be zinc binding [26]. Research has also revealed that two known inducers of ROS, cold exposure and hydrogen peroxide (H_2_O_2_), increase the expression of *MT2a* in *Arabidopsis* [27, 28].

### Phylogeny and structure of ***MeMT*** genes

Phylogenetic analysis assigned *MeMT6, MeMT8* and *MeMT7* to type 2. These *MeMTs* are related to *MTs* in *Arabidopsis*, while *MeMT5*, *MeMT2*, *MeMT1*, and *MeMT3* were assigned to other branches in which the *MTs* are related to those of *Hevea brasiliensis.* These results also revealed that type 2 *MTs* could have different roles in plants. The *MeMT6*, *MeMT7* and *MeMT8* genes have only one intron; in some studies, genes with few or no introns were considered to have enhanced expression levels in plants [29]. To respond to various stresses in a timely manner, genes must be rapidly activated, which would be promoted by a compact gene structure with relatively few introns [30]. This was revealed by *MeMT6*, *MeMT7* and *MeMT8* being more strongly induced under stress than were the other genes (Fig. 6).

### ***MeMT*** genes exhibit tissue-specific expression

The expression patterns of *MT* genes in different tissues have been described in many species, such as *A. thaliana* [6], *T. aestivum* [5], *G. max* [7], *O. sativa* [4] and *S. lycopersicum* [3]. Each type of *MT* exhibits tissue-specific expression. Type 1 *MT* genes are predominantly expressed in both the leaves and the roots, whereas Type 2 *MT* genes are expressed primarily in the leaves, stems, and developing seeds [6, 20, 31]. Type 3 *MT* genes are expressed in the leaves or in ripening fruits [28], and the expression of Type 4 *MT* genes occurs not only in seeds but also in reproductive organs and vegetative tissues [32, 33]. As shown in Fig. 5B, Type 2 *MeMT* genes were highly expressed in the storage roots; the Type 3 *MeMT* genes were highly expressed in the leaves. Taken together, *MeMTs* in the same type have the similar expression patterns.

### Functional characterization of ***MeMTs*** in cassava

MT proteins play a substantial role in response to physiological stress through the regulation of ROS [9, 34–36]. An investigation reported that *MTs* may fundamentally alter redox status and present antioxidant properties against both hydroxyl and peroxyl radicals in sweet potato [37]. Another study showed that under in vivo conditions, *MTs* function as zinc supplies, in which *MTs* release zinc when the degree of responsive nitrogen species (RNS) and ROS levels increase [38]. Zinc-dependent genes have been shown to be involved in the regulation and maintenance of stress tolerance mechanisms in plants[39].

These *MeMTs* were upregulated under H_2_O_2_ treatment and cassava storage roots (0 d, 3 d, 6 d, 9 d), while most *MeMTs* presented low transcript levels under low temperature and wounding treatment.

The study has shown that the accumulation and rapid burst of ROS in cassava storage roots are the main causes of PPD and a reduction in ROS accumulation could delay PPD [40, 41]. In cassava storage roots, the concentrations of H_2_O_2_ were highest at 48 h but then gradually decreased to a low amount [15], the trend of which was similar to that of the SOD and POD activity changes during the PPD process. In this study, the expression of most *MeMTs* increased at all the times in the PPD process, and only a few *MeMT*s decreased in expression at 9 d in the late-stage PPD process. It is known that H_2_O_2_ is considered the major ROS in plants. Our qRT‒PCR results indicated that most of the *MeMTs* could be significantly upregulated by H_2_O_2_ treatment.

### As ROS scavengers, ***MeMTs*** are involved in oxidative stress resistance

Plant *MTs* are involved in oxidative stress resistance, as verified in *O. sativa* [9] and *G. hirsutum* [35]. Moreover, *A. thaliana MT2a* was recently shown to mediate ROS balance during oxidative stress elicited by low temperature [8]. In this study, all four *MeMTs*-silenced cassava plants produced *MeMTs*-silenced leaves with obviously decreased tolerance to H_2_O_2_ and increased lipid peroxidation products (MDA). In the silenced leaves, the reduced expression of *MeMTs* may enhance ROS generation during H_2_O_2_ treatment from two ways, namely a lowered ROS-scavenging capacity and a decreased metal-chelating power [35]. Metal ions can activate NADPH oxidase, the main ROS producer in plant. Since the MeMT activity was significantly reduced by gene silencing, so it can no longer sufficiently protect the plant from H_2_O_2_ stress. Moreover, antioxidant enzyme activity was significantly reduced in the *MeMTs*-silenced leaves, in which the CAT activity, POD activity and APX activity were found to be markedly reduced, increased amounts of ROS accumulated. Thus, silencing *MeMTs* makes the plant possibly more vulnerable to harmfull ROS effects.

## Conclusion

*MTs* play a significant role in regulating ROS associated with the stress response. In this study, we lay a foundation for elucidating the *MeMT*-mediated molecular mechanism underlying plant growth and development as well as stress biology. The gene structure and motif compositions of the proteins were found to be considerably conserved within the same subgroup, and the expression patterns of the *MeMT* genes in different tissues suggested that these genes may have multiple functions. Cis-elements related to hormones and responses to stress are distributed in the promoter regions of *MeMTs*, which leads to differences in responses to abiotic stress. *MeMTs* were silenced by VIGS to evaluate their roles in oxidative stress resistance, the findings of which were consistent with their predicted role as ROS scavengers. This study could serve as a reference for future functional investigations and molecular breeding of cassava.

## Materials and methods

### Plant material and treatments

Unless otherwise noted, cassava genotype SC8 (*Manihot esculenta* Crantz No. SC8) selected for this study was planted at National Cassava Germplasm Repository, Tropical Crops Genetic Resources Institute, Chinese Academy of Tropical Agricultural Sciences, Danzhou City, Hainan Province, from March 2021 to December 2021. To study the relative expression level of *MeMTs* during PPD, the cassava storage roots were stored in an incubator with 26 to 28 °C and 70–80% relative humidity. After 0 d, 3 d, 6 d, and 9 d, the storage roots were collected at each time point, frozen in liquid nitrogen, and then stored at -80 °C until use [15]. To further explore the functions of *MeMTs* by VIGS, the stem cutting of SC8 with 5 buds were cut from 8-month-old cassava plants and planted into plastic pots with nutrient soil and watered regularly [40]. After one month of growth, the plants with numbers of fully developed leaves were used for gene silencing [42]. Cassava genotype SC8 tissue culture seedlings were taken from Tropical Crops Genetic Resources Institute (Haikou, China), which cultured in the tissue culture laboratory, Key Laboratory of Ministry of Agriculture and Rural Affairs for Germplasm Resources Conservation and Utilization of Cassava. For abiotic stress treatment, two-month-old SC8 seedlings were subjected to 20 mM H_2_O_2_ for 6 h, low temperature (4 °C) for 9 d and wounding stress for 6 h [43]. Leaves were removed from the treated plants at each time point for extracting RNA.

### Identification of ***MT*** genes in cassava

The protein sequence of *Manihot esculenta* (*Manihot esculenta* v6, accession: GCA_001659605) were obtained from Ensembl Plants (http://plants.ensembl.org/index.html). The query sequences of *MT* family in *A. thaliana* were download from TAIR database (http://www.arabidopsis.org/). The MT protein sequences of the model plant *Arabidopsis* were used as queries in BLASTP searches against the *Manihot esculenta* genome to identify the corresponding members in cassava [44]. The all putative *MeMT* genes were further confirmed via the Conserved Domain Database (CDD, http://www.ncbi.nlm.nih.gov/cdd/) [45], SMART database (https://smart.embl.de/) [46] and Pfam database (http://pfam-legacy.xfam.org/) [47]. Then, the sequences without Metallotio_2 or Metallothio_PEC domains were discarded. Finally, coding DNA sequence (CDS) length, molecular weight (MW), and isoelectric point (pI) for *MeMTs* were obtained by using ExPasy (http://web.expasy.org/protparam/)[48].

### Phylogenetic analysis and classification of the ***MeMT*** gene family

Multiple sequence alignment of 31 MT proteins from NCBI (https://www.ncbi.nlm.nih.gov/)(Additional file 1: Table S3) was performed using ClustalW (v2.0) [49], and a phylogenetic tree was constructed using the NJ method of MEGA 7.0, with 1000 bootstrap replicates [50].

### ***Cis***-elements predicted in the ***MeMTs*** promoters

The 2 kb upstream sequences of *MeMT* genes were selected as the promoter sequence. PlantCARE software (http://bioinformatics.psb.ugent.be/webtools/plantcare/html/) was used to search the cis-elements [51].

### Gene structure and protein motif analyses

The gene structure and conserved domains were analyzed via TBtools (https://github.com/CJ-Chen/TBtools)[52]. MEME (http://meme-suite.org/) was used for conserved motifs prediction [53].

### Chromosomal distribution and collinearity analysis

The chromosomal positions of the *MeMT* genes were taken from the GFF file, and the location figure was drawn by TBtools [52]. The Dual Synteny Plotter program of TBtools was used to analyze the homology of the *MeMT* genes between cassava and other plant species (including *P. trichocarpa*, *B. rapa*, *V. vinifera*, *A. thaliana*, *O. sativa* and *D. rotundata*).The Ka/Ks value of each gene pair was also calculated by using the KaKs_Calculator2.0 [54].

### Tissue expression analysis of ***MeMTs***

To analyze the expression map of the *MeMT* genes in cassava. RNA-seq data [55] were downloaded from the National Center for Biotechnology Information (NCBI) under BioProject PRJNA324539 (https://www.ncbi.nlm.nih.gov/bioproject/?term=PRJNA324539).

### RNA extraction and qRT‒PCR analysis

Samples (three independent replicates) were collected, immediately stored at -80 °C. RNA extraction and qRT‒PCR was performed as described earlier [1]. The primers of qRT‒PCR were listed in Additional file1: Table S8. The date were obtained from three replicates, and the 2^−△△CT^ method was used to calculated the relative expression levels [56].

### Virus-induced gene silencing in cassava

The pTRV1 and pTRV2 vectors, purchased from NC Biotech company, were used for VIGS in cassava. The partial CDSs of four *MeMTs* (*MeMT4, MeMT8, MeMT9 and MeMT10*) were amplified via PCR and cloned into the pTRV2 vector by using the Nimble cloning method. The Agrobacterium tumefaciens GV3101 cell cultures harboring pTRV2 vector and the recombinant plasmids together with pTRV1 were mixed at a ratio of 1:1 and then infiltrated into cassava leaves with a syringe [57]. After 15 d, the new cassava leaves were used for gene expression, H_2_O_2_ content and enzyme activity assays.

### Determination of H_2_O_2_ and MDA contents

The H_2_O_2_ content and MDA content were measured by commercial assay kits (Grace Biotechnology Company, Jiangsu, China). The details of the methods can be found in the manufacturer’s instructions.

### Determination of antioxidant enzyme activities

The activities of SOD, POD, CAT and APX were measured by commercial assay kits (Grace Biotechnology Company, Jiangsu, China). The details of the methods can be found in the manufacturer’s instructions.

### Statistical analysis

SPSS 16.0. was used for statistical analysis. The data are means ± standard errors (SE) of three independent biological replicates. The values with different letters are significantly differences (*p* < 0.05; n = 3) according to a one-way ANOVA.

## Electronic supplementary material

Below is the link to the electronic supplementary material.

Supplementary Material 1

## Data Availability

All data supporting the conclusions of this article are provided within the article and its additional files. The genomics sequence data of cassava were obtained from Ensembl Plants (http://plants.ensembl.org/index.html). The query sequences of *MT* in *A. thaliana* were download from TAIR database (http://www.arabidopsis.org/).The RNA-Seq data are available in NCBI (https://www.ncbi.nlm.nih.gov/) with the accession number PRJNA324539(https://www.ncbi.nlm.nih.gov/bioproject/?term=PRJNA324539).
